# Monomeric gremlin is a novel vascular endothelial growth factor receptor-2 antagonist

**DOI:** 10.18632/oncotarget.9286

**Published:** 2016-05-11

**Authors:** Elisabetta Grillo, Cosetta Ravelli, Michela Corsini, Kurt Ballmer-Hofer, Luca Zammataro, Pasqua Oreste, Giorgio Zoppetti, Chiara Tobia, Roberto Ronca, Marco Presta, Stefania Mitola

**Affiliations:** ^1^ Experimental Oncology and Immunology, Department of Molecular and Translational Medicine, University of Brescia, Brescia, 25123, Italy; ^2^ Experimental Oncology and Immunology, Department of Molecular and Translational Medicine, National Institute of Neurosciences, University of Brescia, Brescia, 25123, Italy; ^3^ Biomolecular Research, Molecular Cell Biology, Paul Scherrer Institut, Villigen, 5232, Switzerland; ^4^ Center of Genomics Science of IIT@SEMM, Milan, 20139, Italy; ^5^ Glycores2000, Milan, 20155 Italy

**Keywords:** gremlin, BMP, VEGFR2 antagonist, angiogenesis, oligomerization

## Abstract

Angiogenesis plays a key role in various physiological and pathological conditions, including inflammation and tumor growth. The bone morphogenetic protein (BMP) antagonist gremlin has been identified as a novel pro-angiogenic factor. Gremlin promotes neovascular responses via a BMP-independent activation of the vascular endothelial growth factor (VEGF) receptor-2 (VEGFR2). BMP antagonists may act as covalent or non-covalent homodimers or in a monomeric form, while VEGFRs ligands are usually dimeric. However, the oligomeric state of gremlin and its role in modulating the biological activity of the protein remain to be elucidated.

Here we show that gremlin is expressed *in vitro* and *in vivo* both as a monomer and as a covalently linked homodimer. Mutagenesis of amino acid residue Cys141 prevents gremlin dimerization leading to the formation of gremlin^C141A^ monomers. Gremlin^C141A^ monomer retains a BMP antagonist activity similar to the wild-type dimer, but is devoid of a significant angiogenic capacity. Notably, we found that gremlin^C141A^ mutant engages VEGFR2 in a non-productive manner, thus acting as receptor antagonist. Accordingly, both gremlin^C141A^ and wild-type monomers inhibit angiogenesis driven by dimeric gremlin or VEGF-A_165_. Moreover, by acting as a VEGFR2 antagonist, gremlin^C141A^ inhibits the angiogenic and tumorigenic potential of murine breast and prostate cancer cells *in vivo*.

In conclusion, our data show that gremlin exists in multiple forms endowed with specific bioactivities and provide new insights into the molecular bases of gremlin dimerization. Furthermore, we propose gremlin monomer as a new inhibitor of VEGFR2 signalling during tumor growth.

## INTRODUCTION

The transforming growth factor-β (TGF-β) superfamily comprises over thirty cystine-knot secreted proteins, including the members of the bone morphogenetic protein (BMP) family. They transduce a variety of cellular responses through the assembly of heterodimeric serine/threonine kinase receptors, leading to the activation of smad transcription factors [1]. BMPs are aberrantly expressed in multiple malignancies, where they elicit context-dependent effects that can be both detrimental and beneficial for tumor growth and progression [2, 3]. The activity of BMPs is inhibited by various extracellular antagonists that hamper the binding of BMPs to their receptors, including chordin, noggin, follistatin and the DAN (differential screening selected gene aberrative in neuroblastoma) family of BMP antagonists [4–6].

Gremlin is a multifunctional protein belonging to the DAN family of BMP antagonists. It plays essential roles during embryonic development [7–9] and in the pathogenesis of several human diseases, such as fibrosis [10] and cancer [11, 12], where it can promote epithelial-to-mesenchymal transition (EMT) [13] by inhibiting BMP-2, BMP-4 and BMP-7. Also, gremlin, once released by tumor cells, stimulates neovascular and pro-inflammatory responses in a BMP-independent manner [14] due to its capacity to bind and activate vascular endothelial growth factor (VEGF) receptor-2 (VEGFR2), the main transducer of VEGF-mediated angiogenic signals [15–17], and to bind heparan-sulphate proteoglycans (HSPGs) [18, 19] on endothelial cells (ECs). Interestingly, gremlin is overexpressed in several human cancers [12], where it may neutralize the negative regulatory role of BMPs on cell proliferation, modulate EMT [13], and enhance tumor growth by stimulating tumor angiogenesis [14, 15] and inflammation [16].

Beside gremlin, DAN family includes, among others, DAN, cerberus, protein related to DAN and cerberus (PRDC), uterine sensitization-associated gene 1, and sclerostin (SOST). They are small glycosylated proteins characterized by a core ‘DAN’ domain that contains a cystine-knot motif responsible for BMP binding, as shown by NMR analysis of SOST [20]. This suggests that the structure of DAN family members may resemble that of TGF-β superfamily growth factors. Relevant to this point, several TGF-β family members have an unpaired cysteine residue that forms an intermolecular disulphide bridge holding together two monomers and leading to the formation of covalent homo or heterodimers [21]. At variance, PRDC forms highly stable non-covalent homodimers [22] whereas SOST exists as a monomer [20].

Gremlin structure has not been solved yet, however, sequence alignments [21] and homology structural modelling based on human chorionic gonadotropin [23] and SOST [20] predicted that gremlin may form covalent homodimers through Cys141. Recently it has been reported that gremlin may form homodimers [19], but experimental evidences are not available to unambiguously unravel the oligomerization state of gremlin and to correlate it with the biological activities of the protein.

Here we show that gremlin exists both in a monomeric form and as a covalent homodimer held together by Cys141. To understand how gremlin oligomerization affects its biological activity, we compared wild-type gremlin (gremlin^WT^) dimers with a monomeric gremlin mutant where Cys141 was mutated to alanine (gremlin^C141A^). Analysis of the biological activity of gremlin^C141A^ demonstrates for the first time that monomeric gremlin, while retaining its BMP-antagonist activity, is devoid of a significant angiogenic capacity due to its non-productive binding to VEGFR2. Eventually, gremlin monomer acts as a VEGFR2 antagonist, inhibiting the angiogenic activity of dimeric gremlin^WT^ and VEGF-A, and reducing tumor growth and vascularization. In conclusion, our data demonstrate that monomeric gremlin acts as a potent antagonist of VEGFR2, with potential implications in VEGFR2-related physiological and pathological processes, including cancer.

## RESULTS

### Gremlin exists both as a monomer and as a homodimer

**Figure 1:**
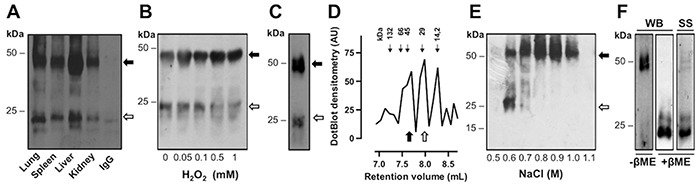
Gremlin exists both as a monomer and a covalent dimer **A.** total lysates from healthy murine organs were immunoprecipitated with anti-gremlin antibody, separated by SDS-PAGE under non-reducing conditions and probed with anti-gremlin antibody. Black arrow, gremlin dimer; open arrow, gremlin monomer. IgG were used as a control. **B.** FGF2-T-MAE cells were treated with increasing concentrations of H_2_O_2_ for 1 hour. At the end of incubation, the cells were incubated for 4 hours with fresh medium. Conditioned medium was collected and immunoprecipitated with anti-gremlin antibody. Immunoprecipitated fractions were analysed by WB under non-reducing conditions. **C-D.** recombinant his-tagged gremlin^WT^ was transiently expressed in HEK293T cells, purified by IMAC and analyzed by SDS-PAGE followed by Western blotting (WB) under non-reducing (C) or by analytical size exclusion chromatography. The elution profile of gremlin was obtained by dot blot analysis of the eluted fractions (D). Black arrows indicate the retention volume of standard proteins (seroalbumin: 132 and 66 kDa; ovalbumin: 45 kDa; carbonic anhydrase: 29 kDa and lactoalbumin: 14.2 kDa). **E.** IMAC purified gremlin was further subjected to heparin-affinity chromatography. Heparin column was washed with a discontinuous NaCl gradient. Eluted fractions were separated by SDS-PAGE under non-reducing conditions and probed with anti-gremlin antibody. Black arrow, gremlin dimer; open arrow, gremlin monomer. **F.** pure dimeric gremlin^WT^ was analysed by SDS-PAGE followed by Western blotting (WB) under non-reducing (−βME) and reducing (+βME) conditions and by silver staining (SS) of the gel.

Gremlin immunoprecipitated from the lysates of murine organs and analysed by SDS-PAGE under reducing conditions migrates as immunoreactive 20-24 kDa bands (data not shown), as anticipated for the non-glycosylated and *N*-glycosylated forms of the protein monomer, respectively [24]. In contrast, when analysed by SDS-PAGE under non-reducing conditions, gremlin is detectable as 20-24 kDa monomers as well as 40-44 kDa immunoreactive bands (Figure 1A), consistent with *in silico* studies predicting gremlin to form covalent homodimers [21, 23]. In the different tissues, the monomer/dimer ratio ranged between 0.8 and 0.5, as assessed by densitometric analysis of the immunoreactive bands. FGF2-transformed murine aortic endothelial cells (FGF2-T-MAE) express gremlin [14] that is released both in monomeric and dimeric forms in the cell culture medium (Figure 1B). In order to understand whether the cellular redox state may affect the gremlin monomer/dimer equilibrium, FGF2-T-MAE cells were treated with H_2_O_2_ and the oligomeric state of gremlin was analyzed under non-reducing conditions. As shown in Figure 1B, H_2_O_2_ treatment induced a dose-dependent increase in the dimer-to-monomer ratio of the released protein, confirming that gremlin may exist in a redox-dependent monomer/dimer equilibrium.

On these bases, his-tagged wild-type gremlin (gremlin^WT^) was expressed in HEK293T cells and purified from the cell supernatant by immobilized metal affinity chromatography (IMAC). Similar to endogenous gremlin, recombinant gremlin is produced and released both in monomeric and dimeric forms (Figure 1C). Size exclusion chromatography demonstrated that IMAC purified gremlin^WT^ elutes in three major peaks with relative retention volumes equal to 7.7, 8.0 and 8.3 mL, consistent with an apparent molecular weight equal to ≈48.3 kDa (dimeric form), ≈25.5 kDa (monomeric form) and ≈13.4 kDa [representing a gremlin breakdown product [25]], respectively (Figure 1D). Thus, gremlin exists in monomeric and dimeric state also under native conditions. In addition, when IMAC purified gremlin^WT^ was further subjected to heparin-affinity chromatography, gremlin dimer eluted from the heparin column at higher ionic strength than the monomer (Figure 1E). On this basis, recombinant gremlin^WT^ dimer could be isolated from its monomer by sequential step-wise elution of the heparin column with 0.6 M and 1.2 M NaCl washes, the latter containing purified gremlin^WT^ uniquely in a dimeric form (98.5% purity as assessed by SDS-PAGE followed by silver staining of the gel) (Figure 1F). Of note, the 13.4 kDa gremlin breakdown product was absent in our preparation after heparin chromatography (Figure 1E–1F).

### Gremlin forms covalently bound homodimers through Cys141

**Figure 2:**
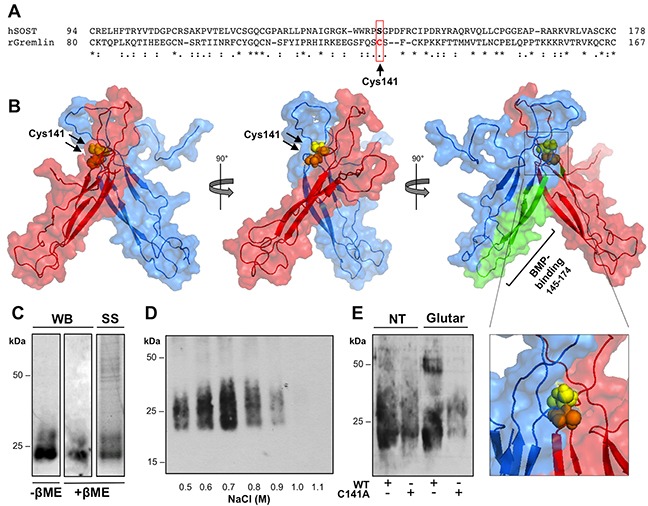
Gremlin forms disulfide-bound homodimers through Cys141 **A.** CLUSTAL W sequence alignment of the cystine-knot domain of gremlin and of structurally related SOST. **B.** surface view of the predicted structure of gremlin homodimer generated by docking simulation with RosettaDock software (model 0267; total score: −29.594; interface score −4.429; interchain contact −20). Gremlin homodimer subunits are in red or light blue. Cys141 residues are in yellow and orange spheres. The previously identified BMP4-binding region of gremlin is depicted in green. **C.** gremlin^C141A^ mutant was transiently expressed in HEK293T cells and sequentially purified from cell supernatant by IMAC and heparin-affinity chromatography. Heparin column was eluted with a discontinuous NaCl gradient. Eluted fractions were separated by SDS-PAGE under non-reducing conditions and probed with anti-gremlin antibody. **D.** purified gremlin^C141A^ monomer was analysed by WB under non-reducing and reducing conditions and by SS of the gel. **E.** Gremlin ^wt^ and gremlin^C141A^ were incubated in the absence or in the presence of 0.05% glutaraldehyde for 20 minutes at RT, separated by SDS-PAGE under reducing conditions and probed with anti-gremlin antibody.

*In silico* studies predicted that gremlin may form covalent homodimers through a Cys141-Cys141 disulfide bridge [21]. This hypothesis is supported by the observed effect of the intracellular redox state on the dimer-to-monomer ratio of the released protein (see above) and with the absence of the corresponding Cys residue in monomeric SOST [20] (Figure 2A). On this basis, RosettaDock software [26] was applied for *in silico* docking of two gremlin monomers whose conformation was obtained by homology modelling using the NMR structure of SOST as a template (ModBase: 4E2EED0731DA83D06F0DFD6B8C55B387) [20]. Among the 10 top scoring models generated by RosettaDock software, the model shown in Figure 2B (model ID: 0267; total score: −29.594; interface score −4.429; interchain contact −20) shows that Cys141 residues are located at the interface between the two monomers at a distance compatible with an intermolecular disulfide bridge.

To assess this hypothesis, we generated a recombinant gremlin mutant in which Cys141 was replaced by an Ala residue (gremlin^C141A^). Similar to gremlin^WT^, his-tagged gremlin^C141A^ was expressed and purified from HEK293T cell supernatant by sequential IMAC and heparin affinity chromatography (85.9% purity). As anticipated, gremlin^C141A^ does not form covalent homodimers, only 23-27 kDa immunoreactive bands being detectable after SDS-PAGE under non-reducing conditions (Figure 2C). Similarly to gremlin^WT^ monomer, gremlin^C141A^ binds to the heparin column with an affinity lower than gremlin^WT^ dimer, eluting from the column at 0.6-0.7 M NaCl (Figure 2D). Moreover glutaraldehyde treatment rules out the possibility that gremlin^C141A^ may form non covalent dimers (Figure 2E), while confirming that gremlin^WT^ exists in a dimeric form.

### Monomeric gremlin^C141A^ retains its BMP antagonist activity

**Figure 3:**
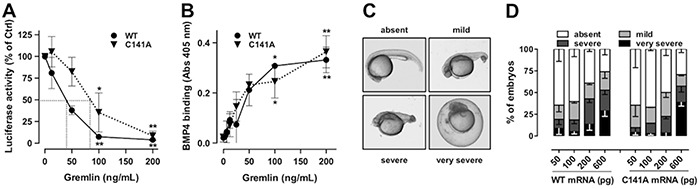
GremlinC141A retains its BMP-antagonist activity **A.** HepG2 cells were transiently transfected with a construct harbouring BMP-responsive hepcidin promoter upstream to the luciferase reporter gene. Serum-starved transfected cells were then stimulated with 50 ng/mL of BMP4 in the absence or the presence of increasing amounts of either gremlin^WT^ (•) or gremlinC141A (▼). After 16 hours incubation, cells were lysed, and luciferase activity was measured. Data are expressed as percent of luciferase activity measured in BMP4 stimulated cells and are the mean ± SEM of 3 independent experiments. Two-Way ANOVA followed by Bonferroni's test revealed that the dose-response curves of the two proteins were not statistically different. **B.** 96-well plates coated with 250 ng/mL of BMP4 were incubated with increasing concentrations of gremlin^WT^ or gremlin^C141A^. Then, the capacity of gremlin^WT^ (•) or gremlin^C141A^ (▼) to bind to immobilized BMP4 was assessed by ELISA assay using an anti-gremlin antibody. **C-D.** zebrafish embryos were injected (at 1-4 cell stage) with the indicated doses of either gremlin^WT^ or gremlin^C141A^ mRNA. Representative dorsalized embryo phenotypes at 24 hours after injection (C). All embryos were viewed laterally. D, percentage of embryos in different categories at 24 hours after injection. Data are the mean ± SEM of 3 independent experiments. *, p<0.05; **, p<0.01, One-Way ANOVA followed by Bonferroni's test versus control.

BMP antagonists bind to and sequester BMPs in inactive complexes, preventing their interaction with cognate receptors and the transcription of various BMP-dependent genes, including hepcidin [27]. On this basis, to assess how gremlin oligomerization might affect its BMP antagonist activity, purified gremlin^WT^ dimer and gremlin^C141A^ monomer were evaluated for their capacity to inhibit the BMP4-dependent up-regulation of the luciferase reporter gene driven by the BMP-responsive hepcidin promoter in HepG2 cells [27]. As shown in Figure 3A, the two proteins inhibit BMP4 activity with similar potency, with ID_50_ values equal to 40 and 80 ng/mL, respectively. Accordingly, dose-response ELISA experiments demonstrated that gremlin^WT^ dimer and gremlin^C141A^ monomer bind immobilized BMP4 with a similar capacity (Figure 3B), thus confirming the presence of similar amounts of active molecules per unit mass in the two protein preparations.

BMPs control dorso-ventral symmetry during embryonic development and the overexpression of BMP antagonists results in dorsalized embryos [28]. In order to further assess the effect of oligomerization on the BMP antagonist activity of gremlin, gremlin^WT^ and gremlin^C141A^ mRNAs were injected at the 1-4 cell stage in zebrafish embryos and their effect on dorsalization was assessed at 24 hours after injection. As shown in Figure 3C, embryos were scored as normally developed embryos or according to the severity of their dorsalization defects: “mild phenotype” embryos showed a smaller posterior trunk with a curly tail and shortened yolk extension; “severe phenotype” embryos had visible head structures, but curly trunk and tail; “very severe phenotype” embryos had undistinguishable tissue structures sitting on the yolk ball and could not be de-chorionated. Increasing doses of gremlin^WT^ and gremlin^C141A^ mRNAs produced comparable dorsalization outcomes, confirming their similar BMP antagonist effects (Figure 3D).

### Monomeric gremlin^C141A^ is devoid of pro-angiogenic activity

**Figure 4:**
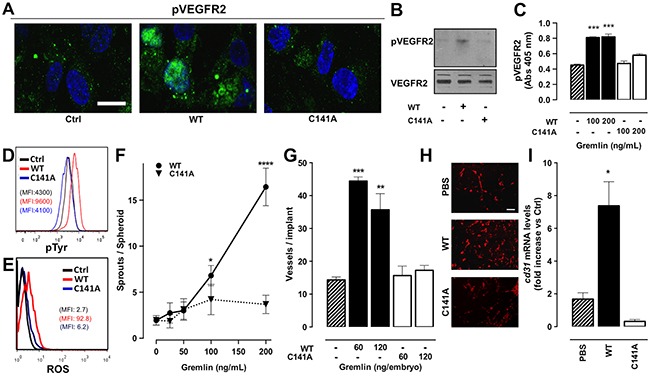
Monomeric gremlinC141A is devoid of pro-angiogenic activity **A-C.** serum-starved HUVECs were treated for 5 minutes with 100 ng/mL (or the indicated dose) of gremlin^WT^ or gremlin^C141A^. Treated cells were then immunostained with anti-phospho-VEGFR2 (Y1175) antibody followed by Alexa Fluor 488 secondary antibody and nuclear counterstaining with DAPI (scale bar, 10 μm) (A) or by HRP secondary antibody (B). Uniform loading of the gels was confirmed by incubation of the membranes with anti-VEGFR2 antibodies. Alternatively, the capacity of gremlin^WT^ (black bars) or gremlin^C141A^ (open bars) to induce VEGFR2 phosphorylation was assessed by ELISA assay, using anti-pVEGFR2 (Y951) antibody (C). Data are the mean ± SEM of 3 independent experiments. **D.** VEGFR2-overexpressing ECs were stimulated for 10 minutes with 20 ng/mL of gremlin^WT^ (red line) or gremlin^C141A^ (blue line) and assessed by FACS analysis for total tyrosine phosphorylation using an anti-phospho-Tyr antibody. **E.** FACS analysis of ROS production was carried out on HUVEC pre-loaded with 5 μM DCFH-DA and stimulated for 10 minutes with 10 ng/mL of gremlin^WT^ (red line) or gremlin^C141A^ (blue line). Representative mean fluorescence intensity histograms are shown. **F.** HUVEC spheroids, embedded in fibrin gel, were stimulated with increasing concentrations of gremlin^WT^ (•) or gremlin^C141A^ (▼). After 24 hours EC sprouts were counted. Data are the mean ± SEM of the number of sprouts/spheroid measured in 50 spheroids. **G.** alginate implants containing 60-120 ng of gremlin^WT^ (black bars) or gremlin^C141A^ (open bars) were grafted onto chicken embryo CAMs. After 72 hours, neovessels converging towards the implant were counted. Data are expressed as mean ± SEM (n=10). **H-I.** liquid Matrigel was mixed with 1.0 μg/mL gremlin^WT^ or gremlin^C141A^ and injected subcutaneously into the flank of C57BL/6 mice. Matrigel with PBS alone was used as negative control. One week after injection, plugs were harvested and CD31+ endothelial cells were examinated by immunofluorescence (H, scale bar, 10 μm) and *cd31* mRNA expression levels were measured by RT-qPCR analysis (I). Data are the mean ± SEM (n=10-15) and are expressed as relative expression ratios (ΔΔCt– Fold increase) using one PBS plug as reference. *, p<0.05; **, p<0.01; ***, p<0.005; ****, p<0.001, One-Way ANOVA followed by Bonferroni's test versus control.

Gremlin is a non-canonical angiogenic VEGFR2 agonist [14, 15]. To assess whether monomeric gremlin^C141A^ retains a significant pro-angiogenic activity, we evaluated its capacity to activate VEGFR2 in HUVECs. Immunofluorescence, Western blot and ELISA analyses revealed that gremlin^C141A^ does not induce a significant VEGFR2 phosphorylation when compared to gremlin^WT^ (Figure 4A–4C), although the two preparations contain similar amounts of active molecules per unit mass, as indicated by their similar BMP antagonist potency. In addition, FACS analyses demonstrated that gremlin^C141A^ is unable to induce a significant increase of total tyrosine phosphorylation and ROS production in both VEGFR2-overexpressing ECs and HUVEC when compared to gremlin^WT^ (Figure 4D and 4E). Accordingly, monomeric gremlin^C141A^ fails to trigger the formation of endothelial sprouts from HUVEC spheroids embedded in fibrin gel (Figure 4F).

In keeping with the *in vitro* observations, gremlin^C141A^ is devoid of a significant angiogenic activity *in vivo* in the chick embryo chorioallantoic membrane (CAM) assay (Figure 4G). Also, gremlin^C141A^ does not induce neovessel formation in a murine Matrigel plug assay, as assessed by immunofluorescence analysis using anti CD31 antibodies (Figure 4H) and by RT-qPCR analysis of *cd31* mRNA levels in the plugs (Figure 4I). At variance, dimeric gremlin^WT^ induces angiogenic responses in all these assays.

### Monomeric gremlin^C141A^ exerts a non-productive interaction with VEGFR2

**Figure 5:**
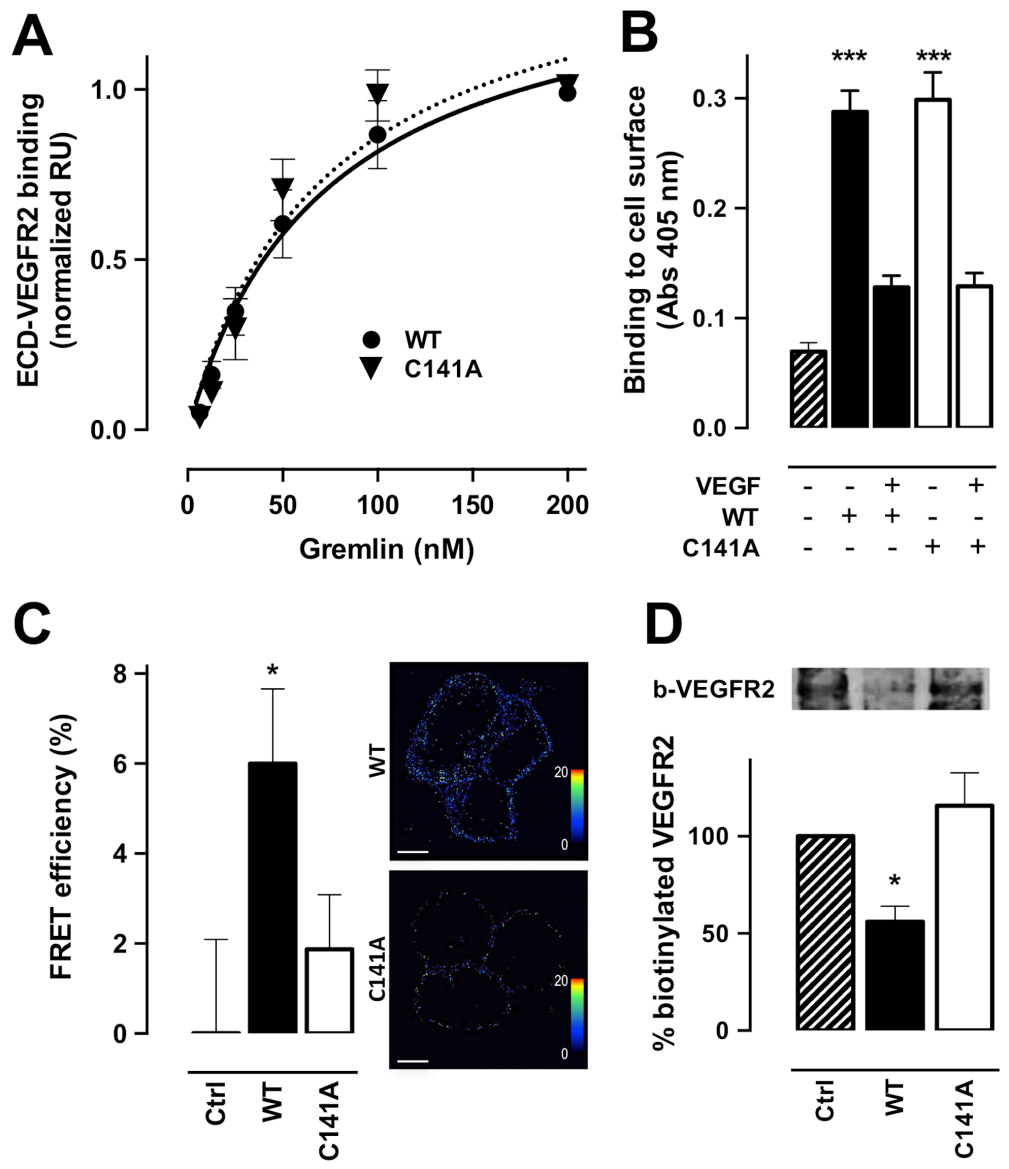
Monomeric gremlinC141A exerts a non-productive interaction with VEGFR2 **A.** increasing concentrations of gremlin^WT^ or gremlin^C141A^ were injected on ECD-VEGFR2-coated CM5 sensorchips. Response units (RU) were recorded as a function of time. For each concentration of the ligand, the SPR response at equilibrium was used to build the normalized dose-response binding isotherms of gremlin^WT^ (•) and gremlin^C141A^ (▼) with ECD-VEGFR2. Data are the mean ± SEM of 2 independent experiments. The continuous and dashed lines are the Langmuir fits for the data points of gremlin^WT^ and gremlin^C141A^ respectively. **B.** VEGFR2-overexpressing ECs were incubated with 150 ng/mL of gremlin^WT^ (black bar) or gremlin^C141A^ (open bar) in the absence or presence of 1.5 μg of VEGF-A_165_ for 2 hours at 4°C. Cells were then washed with 2.0 M NaCl to remove gremlin bound to HSPGs and gremlin bound to cell-surface VEGFR2 was revealed by ELISA using anti-gremlin antibody. Data are the mean ± SEM of 3 independent experiments. **C.** HEK293T cells were transiently co-transfected with CFP-tagged and YFP-tagged ECD-VEGFR2 and stimulated with 100 ng/mL of gremlin^WT^ (black bars) or gremlin^C141A^ (open bars) for 10 minutes. Cells were then analysed for FRET efficiency by acceptor photo-bleaching. FRET efficiency was calculated using the formula: FRET = (D_post_ − D_pre_)/D_post_, where D_post_ and D_pre_ represent the donor (ECFP) emission intensities before and after photo bleaching, respectively. Representative pictures of FRET efficiency are shown in right panels, where dots correspond to FRET events (rainbow colour: range 0-20 FRET efficiency). Data are the mean ± SEM (n=20). **D.** HUVECs were stimulated with 100 ng/mL of gremlin^WT^ (black bars) or gremlin^C141A^ (open bars) for 15 minutes at room temperature. Cell-surface proteins were then labelled with biotin 3-sulfo-N-hydroxysuccinimide ester sodium salt. Cell lysates were immunoprecipitated with anti-VEGFR2 antibody, separated by SDS-PAGE and probed with HRP-streptavidin to visualize biotinylated VEGFR2 (upper panel). Densitometric analyses were performed on blots obtained from 3 independent experiments and data are expressed as % of control (bottom panel). *, p<0.05; *** p<0.005, One-Way ANOVA followed by Bonferroni's test versus control.

Surface plasmon resonance (SPR) analysis had shown that gremlin and VEGF-A_165_ bind the extracellular domain (ECD) of VEGFR2 with similar affinities [29]. On this basis, we assessed the capacity of gremlin^C141A^ to bind ECD-VEGFR2 immobilized to a BIAcore sensorchip. Surprisingly, monomeric gremlin^C141A^ and dimeric gremlin^WT^ bind ECD-VEGFR2 with the same affinity (K_d_ = 73 ± 35 nM and 73 ± 15 nM, respectively) (Figure 5A). In keeping with SPR results, the two proteins equally bind to high affinity sites on the cell surface of VEGFR2-overexpressing ECs, as assessed by ELISA (Figure 5B). Of note, gremlin binding is totally competed by VEGF-A_165_. Moreover, FRET analysis performed on HEK293T cells co-transfected with CFP-tagged and YFP-tagged ECD-VEGFR2 showed the formation of CFP-ECD-VEGFR2/YFP-ECD-VEGFR2 complexes upon stimulation with gremlin^WT^ dimer but not with gremlin^C141A^ monomer (Figure 5C). In addition, cell surface biotinylation analysis demonstrated that, at variance with gremlin^WT^, gremlin^C141A^ does not induce VEGFR2 internalization in HUVECs (Figure 5D). Taken together, our data demonstrate that monomeric gremlin^C141A^ can bind VEGFR2, but is devoid of the capacity to induce receptor dimerization and internalization, leading to a non-productive interaction with the receptor.

### Monomeric gremlin is a VEGFR2 antagonist

**Figure 6:**
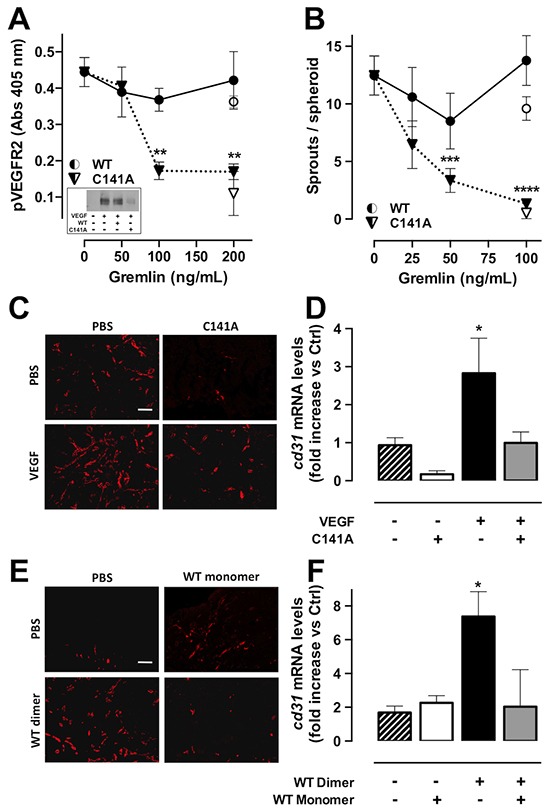
GremlinC141A is a VEGFR2 antagonist **A.** serum-starved HUVECs were stimulated with 5 ng/mL of VEGF-A_165_ for 10 minutes in the absence or the presence of increasing concentrations of gremlin^WT^ (•) or gremlin^C141A^ (▼). pVEGFR2 (Y951) levels were then assessed by ELISA. Data are the mean ± SEM of 3 independent experiments. Gremlin^WT^ (○) or gremlin^C141A^ (▽) alone were used as controls. VEGFR2 phosphorylation was confirmed by WB analysis using anti-pVEGFR2 (Y1175) antibody (**inset**). **B.** HUVEC spheroids, embedded in fibrin gel, were stimulated with 10 ng/mL of human VEGF-A_165_ in the presence of increasing concentrations of gremlin^WT^ (•) or gremlin^C141A^ (▼). Gremlin^WT^ (○) or gremlin^C141A^ (▽) alone were used as controls. After 24 hours, EC sprouts were counted. Data are the mean ± SEM of the number of sprouts/spheroid measured in 50 spheroids. **C-D.** VEGF-A_165_ (1.0 μg/mL) was mixed with liquid Matrigel in the absence (black bar) or the presence of gremlin^C141A^ (5.0 μg/mL, grey bar) and injected subcutaneously in the flank of C57BL/6 mice. Plugs containing PBS or gremlin^C141A^ (open bar) alone were used as controls. One week after injection, plugs were harvested and CD31+ endothelial cells were examinated by immunofluorescence (C, scale bar, 10 μm) and *cd31* mRNA expression levels were measured by RT-qPCR analysis (D). **E-F.** Liquid Matrigel was mixed with 400 ng/mL of monomeric gremlin^WT^ (open bar) or dimeric gremlin^WT^ (black bar) or with 800 ng/mL of a sample containing both gremlin^WT^ monomer and dimer (relative amounts: 46% and 54%, respectively; grey bar). Then, plugs were injected subcutaneously into the flank of C57BL/6 mice. Plugs containing PBS were used as controls. One week after injection plugs were harvested and and CD31+ endothelial cells were examinated by immunofluorescence (E, scale bar, 10 μm) and *cd31* mRNA expression levels were measured by RT-qPCR (F). *, p<0.05; **, p<0.01; ***, p<0.005; ****, p<0.001, One-Way ANOVA followed by Bonferroni's test versus control.

To assess whether gremlin^C141A^ may act as a VEGFR2 antagonist, we evaluated the capacity of gremlin^C141A^ to affect the pro-angiogenic of activity of VEGF-A_165_. To this purpose, HUVECs were treated with VEGF-A_165_ in the absence or in the presence of increasing concentrations of gremlin^C141A^. Monomeric gremlin^C141A^ inhibits VEGFR2 phosphorylation and the sprouting of HUVEC spheroids triggered by VEGF-A_165_ in a dose-dependent manner (Figure 6A–6B). Under the same experimental conditions, gremlin^WT^ does not affect the activity of VEGF-A_165_.

To evaluate whether gremlin^C141A^ may hamper the angiogenic potential of VEGF-A_165_ also *in vivo*, Matrigel plugs containing 400 ng of VEGF-A_165_ were injected subcutaneously in mice in the absence or in the presence of 2.0 μg of gremlin^C141A^. Matrigel containing PBS or gremlin^C141A^ alone were used as controls. In keeping with the *in vitro* results, gremlin^C141A^ significantly reduces the angiogenic activity of VEGF-A_165_ also *in vivo*, as assessed by immunofluorescence analysis using anti CD31 antibodies and RT-qPCR analysis of *cd31* mRNA levels in the plugs (Figure 6C–6D). Taken together, our results show that gremlin^C141A^ abrogates the angiogenic activity of VEGF-A_165_ by acting as a VEGFR2 antagonist. To evaluate whether this capacity is limited to the monomeric gremlin^C141A^ mutant or it is shared also by the gremlin^WT^ monomer, gremlin^WT^ dimer and a mixture containing equivalent amounts of monomeric and dimeric gremlin^WT^ were assessed for their angiogenic activity in the Matrigel plug assay. Similar to monomeric gremlin^C141A^, monomeric gremlin^WT^ is devoid of a significant pro-angiogenic capacity and fully abolishes the angiogenic activity of dimeric gremlin^WT^ (Figure 6E–6F).

### Monomeric gremlin inhibits tumor growth and angiogenesis

**Figure 7:**
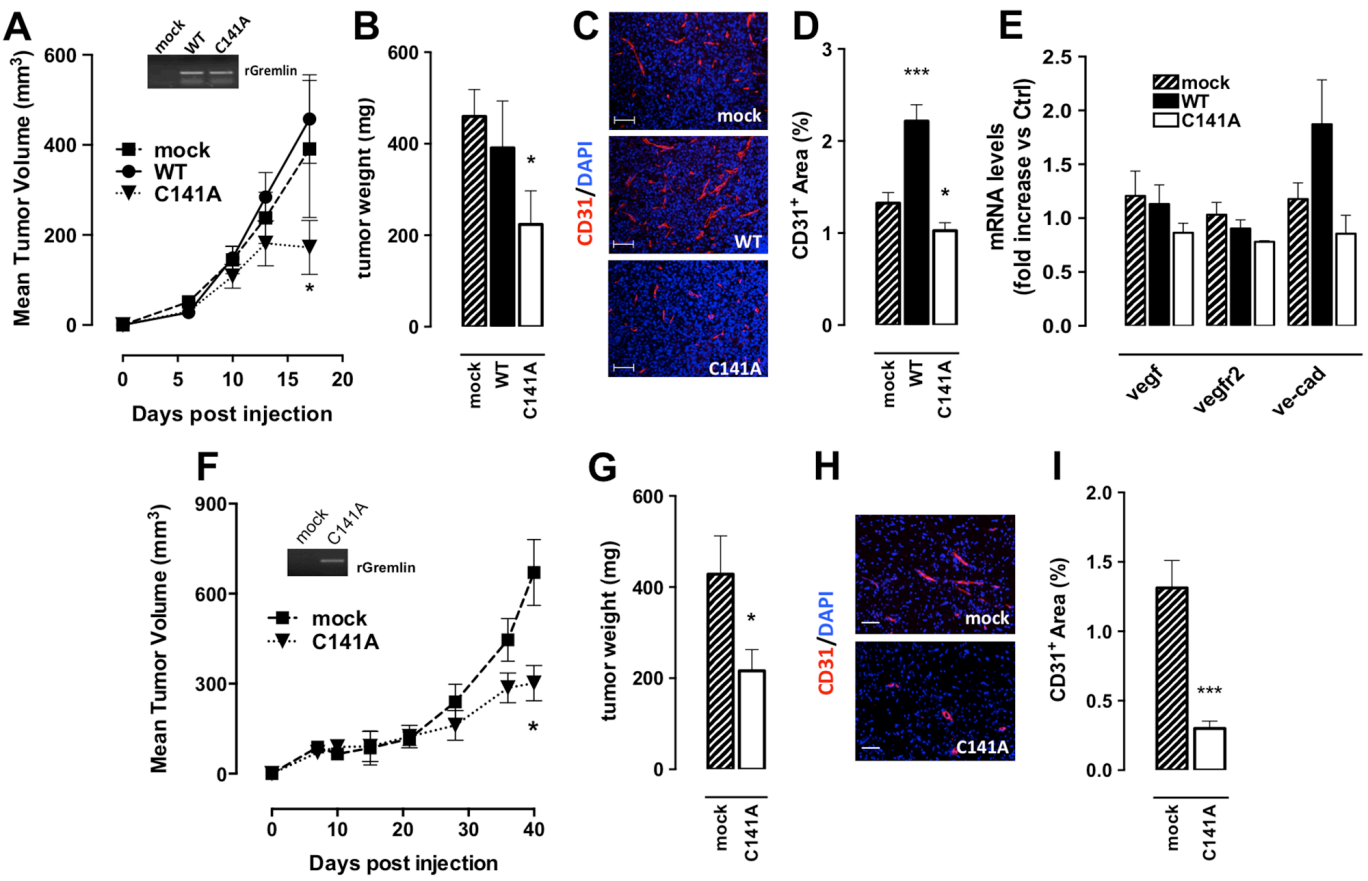
GremlinC141A reduces the tumorigenic and angiogenic potential of EO771 cells **A.** 5×10^5^ mock (▪)- or gremlin^WT^ (•)- or gremlin^C141A^ -EO771 (▼) cells were injected orthotopically in 15-week-old female mice (6 mice/group). Tumor growth was followed for up to 17 days. Tumor volume (in mm^3^) is expressed as a function of time. In the inset the PCR analysis for exogenous rat gremlin mRNA in tumor samples is shown. At the end of experiments tumors were harvested, weighed **B** and analysed by immunofluorescence using anti CD31 antibodies (red). Nuclei were counterstained with DAPI (blue) (scale bar 100 μm) **C. D.** CD31^+^ area was quantified and normalized to area (3-5 fields per tumor). Data are the mean ± SEM. **E.** murine *vegf*, *vegfr2* and *ve-cadherin* (*ve-cad*) mRNA expression levels were measured by RT-qPCR. Data were normalized on murine *gapdh* and are expressed as fold increase vs mock tumors. **F.** 4×10^6^ mock (▪)- or gremlin^C141A^ TRAMP-C2 (▼) cells were injected subcutaneously in 15-week-old male mice (6 mice/group). Tumor growth was followed for up to 40 days. Tumor volume (in mm^3^) is expressed as a function of time. In the inset the PCR analysis for exogenous rat gremlin mRNA in tumor samples is shown. At the end of experiments tumors were harvested, weighed **G** and vessel density was analysed by immunofluorescence using anti CD31 antibodies **H.** CD31^+^ area was quantified and normalized to area (3-5 fields per tumor). Data are the mean ± SEM **I.** *, p<0.05; ***, p<0.005, One-Way ANOVA followed by Bonferroni's test versus control.

The activation of VEGFR2 signalling represents a hallmark of tumor angiogenesis. Indeed, inhibitors targeting the VEGF-A/VEGFR2 axis strongly hamper tumor neovascularization and are part of numerous therapeutic protocols for the treatment of cancer patients [30, 31]. In order to assess the effect of monomeric gremlin^C141A^ on tumor angiogenesis, murine VEGF-dependent breast cancer EO771 cells [32, 33] were stably transfected with gremlin^WT^ or gremlin^C141A^ cDNAs and injected orthotopically in syngeneic C57BL/6 female mice. Tumor growth was followed for up to 17 days and then tumors were harvested and processed for RT-qPCR and immunofluorescence analyses. As shown in Figure 7A and B, gremlin^C141A^ overexpression caused a significant inhibition of tumor growth compared with mock tumors. Of note, gremlin^WT^ and gremlin^C141A^ overexpression did not alter the tumor expression levels of *vegf* and *vegfr2*, as assessed by qPCR analysis of harvested grafts (Figure 7E). In addition, as observed for EO771-derived tumors treated with the VEGFR tyrosine kinase inhibitor SU112248 [33], gremlin^C141A^ overexpression efficiently inhibited tumor neovascularization, as demonstrated by the reduction of *ve-cadherin* mRNA levels and CD31^+^ area in gremlin^C141A^-EO771 tumors when compared to mock lesions (Figure 7C–7E). At variance, gremlin^WT^-overexpression increased the neovascularization of gremlin^WT^-lesions.

In keeping with these observations, gremlin^C141A^ overexpression inhibits the growth and vascularization of tumors induced by murine prostate adenocarcinoma TRAMP-C2 cells [34] injected subcutaneously in syngeneic C57BL/6 male mice (Figure 7F–7I). Together, these data indicate that monomeric gremlin^C141A^ inhibits the growth and vascularization of VEGF-dependent tumors by acting as a VEGFR2-antagonist.

## DISCUSSION

Accumulating evidences indicate that gremlin is a multifunctional protein involved in several physiological and pathological processes, including embryonic development, neurodegeneration, inflammation and angiogenesis [35–37]. Gremlin is a member of the TGF-β superfamily, initially characterized as a BMP antagonist. BMP antagonists are expressed as covalently linked homodimers [like activin [4], noggin [5] and BMPs [21]], highly stable non-covalent homodimers [PRDC [22]] or monomers [follistatin [4] and SOST [20]]. Gremlin also elicits potent BMP-independent angiogenic responses by acting as a non-canonical VEGFR2 ligand [15]. At variance with BMP antagonists, VEGFR ligands need to be in a dimeric form in order to fully activate their cognate receptors and exert their pro-angiogenic function [38]. The presence of multiple oligomeric states is occasionally found among TK receptor ligands [39], including VEGFR ligands [40–43], and represents a mechanism of modulation of their activity. Unique among BMP-antagonists, here we show that gremlin exists both as a monomer and as a covalent homodimer in a monomer/dimer equilibrium *in vitro* and *in vivo*.

*In silico* docking predicted that amino acid residue Cys141 may mediate gremlin homodimerization via the formation of a disulfide bond between two protomers. In our model, which differs from the recently proposed head-to-tail homology model of gremlin homodimer based on PRDC structure [19], gremlin protomers dimerize with a head-to-head orientation, forming a “clamp-shaped” dimer with two extended “clip” segments, similar to noggin [5]. In keeping with the structure of noggin/BMP7 complex [5], our model shows that the previously identified BMP-binding motif, corresponding to amino acid sequence gremlin (145-174) [23], spans along the “clip” segments, being available for BMP binding. Site-directed Cys141-to-Ala mutagenesis originated the gremlin^C141A^ mutant that, at variance with gremlin^WT^, is secreted only in a monomeric form. Taken together, our results unequivocally demonstrate for the first time the covalent nature of gremlin homodimers, unravelling the key role of Cys141 in the covalent homodimerization of gremlin protomers.

The molecular bases of the interaction of BMP antagonists with their ligands are poorly understood. As anticipated, BMP antagonists exists both in monomeric and dimeric states [5, 20]. Thus, the activity of BMP antagonists does not appear to be strictly dependent on their covalent dimerization. Here we show that gremlin can function as a BMP antagonist both in a monomeric and dimeric state, gremlin^C141A^ monomer and gremlin^WT^ dimer being equally effective in binding BMP4 and inhibiting BMP-mediated biological responses *in vitro* and in zebrafish embryos. It must be pointed out that the capacity of gremlin^C141A^ to bind BMP4 with an affinity similar to gremlin^WT^ indicates that the lack of homodimerization cannot be the mere consequence of significant alterations of the conformation of the mutagenized protein.

Productive binding to VEGFRs requires the ligand to be in a dimeric state. Indeed, VEGF-A, VEGF-B and PlGF are all active as disulfide-linked homodimers [38] and VEGF-C and VEGF-D exist in a monomer/dimer equilibrium [40–43], variants with enhanced dimer stability showing a stronger activity. Also, recombinant VEGF heterodimers, in which only one of the two protomers retains a receptor binding capacity, exert a potent antagonist activity by preventing VEGFR dimerization [44, 45]. Here we show that monomeric gremlin^C141A^ binds VEGFR2 with the same affinity as dimeric gremlin^WT^. However, the interaction is not productive and fails to trigger receptor dimerization and internalization. Accordingly, gremlin^C141A^ is unable to elicit VEGFR2-dependent angiogenic responses *in vitro* and *in vivo*.

Similar to monomeric VEGF [46], gremlin^C141A^ monomer prevents the angiogenic activity of VEGF-A as well as of gremlin^WT^ dimer, acting as a VEGFR2 antagonist. The VEGFR2-antagonist capacity of monomeric gremlin^C141A^ results in the reduction of tumor growth and vascularization in breast carcinoma and prostatic cancer models. Therefore, our data show that gremlin monomer has unique biological properties that allow for the first time the dissociation of the BMP antagonist activity of gremlin from its VEGFR2-dependent functions, representing a valuable tool for the elucidation of gremlin biology in physiological and pathological settings.

Here we provide evidences that the modulation of the oligomeric state of gremlin allows to separate the receptor binding activity of gremlin from its VEGFR2 dimerization activity, thus enabling the design of receptor antagonists. Because aberrant VEGFR2 signalling is associated with tumor neovascularization, targeting VEGFR2 activation has become an intense area of anti-cancer research. The mutated monomeric variant of gremlin described in this report may provide the basis for the development of new VEGFR2-specific strategies to inhibit pathological angiogenesis.

Gremlin is expressed in several tumors by stromal and parenchymal cells, including activated endothelial cells [12, 14, 47]. BMPs elicit effects that can be either detrimental or beneficial for tumor growth and progression [2]. In addition, BMPs induce endothelial cell proliferation and migration *in vitro* and angiogenesis *in vivo* [3, 48]. Thus, gremlin and other extracellular BMP antagonists have been alternatively considered tumor promoting or oncosuppressive factors. To this regard, it must be pointed out that monomeric wild-type gremlin recapitulates the antagonistic activity of the monomeric gremlin^C141A^ mutant. This suggests that tuning of the monomer/dimer equilibrium may modulate VEGFR2 activation or inhibition by gremlin, the oligomerization state of the protein representing a possible post-translational mechanism of control of the activity of the highly angiogenic dimer in physiological and pathological conditions, including cancer. In keeping with this hypothesis, our data demonstrate that the redox status of the cell affects gremlin dimerization, as observed for VEGF-C [42] and VEGF-D [43]. Thus, the redox microenvironment may modulate the monomer/dimer equilibrium of gremlin during tumor growth, resulting in either a pro- or anti-angiogenic phenotype because of the balance between the VEGFR2 agonist and antagonist activity of the protein. Accordingly, our data show that gremlin^WT^ overexpression results in a further increase of blood vessel density in VEGF-dependent EO771 tumor grafts, whereas overexpression of the monomeric mutant causes a significant decrease of tumor vascularization and growth.

In conclusion, we demonstrate that gremlin functions both as a monomer and as a covalent homodimer and that dimerization is strictly required for the pro-angiogenic activity of gremlin, being dispensable for its BMP antagonist activity. In addition, we show that monomeric gremlin is a potent VEGFR2 antagonist endowed with anti-angiogenic, anti-tumor functions. Our findings provide new insights into the molecular bases of gremlin dimerization with significant consequences for its biological activity. Monomeric gremlin^C141A^ represents as a novel VEGFR2 antagonist that deserves further attention to disclose potential clinical implications for the treatment of VEGF-dependent tumors.

## MATERIALS AND METHODS

### Cell cultures

Human embryonic kidney HEK 293T/17 (ATCC) cells, human hepatocellular carcinoma HepG2 (ATCC) cells, fetal bovine aortic endothelial GM7373 cells [49], FGF2-T-MAE cells [14] and murine breast cancer EO771 cells (kindly provided by R.Giavazzi, Mario Negri, Italy) were grown in Dulbecco modified Eagle's medium (DMEM, Gibco Life Technologies) containing 10% fetal calf serum (FCS, Gibco). Murine prostate adenocarcinoma TRAMP-C2 cells, obtained from ATCC-LGC Standards Repository (ATCC No CRL-2731) were grown in maintained in DMEM supplemented with 10% heat inactivated FCS, 10 mM HEPES Buffer, 0.5 mM 2-mercaptoethanol, 2.0 mM glutamine, 5 mg/L bovine insulin (Sigma-Aldrich) and 10 nM DHT. GM7373 cells were stably transfected with pcDNA3.1 vector (Invitrogen Life Technologies), harbouring mouse VEGFR2 cDNA to generate VEGFR2-overexpressing ECs. EO771 and TRAMP-C2 cells were stably transfected with empty pcDNA3 vector or pcDNA3 plasmid harbouring rat gremlin^WT^ or mutant gremlin^C141A^. Human umbilical vein endothelial cells (HUVECs) were grown in M199 medium (Gibco Life Technologies) supplemented with 20% fetal calf serum (FCS, Gibco Life Technologies), endothelial cell growth factor (100 μg/ml) (Sigma Chemical Co., St. Louis, MO) and porcine heparin (Sigma) (100 μg/ml). HUVECs were used at early passages (I-IV) and grown on plastic surface coated with porcine gelatin (Sigma).

### Generation of DNA constructs

The cDNA sequence of rat gremlin^WT^ was amplified from pMEX-rGremlin (NM_019282.3) [provided by L. Topol [50]] and cloned in pcDNA3 vector (Invitrogen) upstream to His-Tag sequence to obtain pcDNA3-rGremlin-HisTag^WT^. Mutagenesis of Cys141-to-Ala was carried out using the QuikChange Lightning Site-directed Mutagenesis Kit (Agilent Technologies). Primers forward 5′-CCTTTCAGTCCGCCTCCTTCTGCAAGCCC-3′ and reverse 5′-CTTGCAGAAGGAGGCGGACTGAAAGGAACC-3′ were used to obtain pcDNA3-rGremlin-HisTag^C141A^.

### Recombinant gremlin expression and purification

His-tagged recombinant gremlin^WT^ and gremlin^C141A^ were transiently expressed in HEK293T cells. HEK293T cells were transfected using polyethylenimine (PEI, Polysciences, Inc.) in DMEM supplemented with 0.5% FCS. 12 hours after transfection, the medium was collected, clarified and dialysed against 5.0 L of 20 mM phosphate buffer (pH 8.0) containing 0.5 M NaCl (binding buffer). Gremlin was purified from the dialysed medium by IMAC on 1.0 mL HiTrap Chelating HP Ni^2+^-column (GE Healthcare), pre-equilibrated with binding buffer, using AKTA™ start FPLC system (GE Healthcare). The column was eluted with a discontinuous imidazole gradient (10, 40 and 250 mM), gremlin eluting at 250 mM imidazole. Partially purified gremlin was buffer exchanged with PBS and subjected to heparin-affinity chromatography. To this purpose, IMAC purified gremlin was loaded onto an heparin-Sepharose column, which was then eluted with a discontinuous NaCl gradient (0.3-1.2 M NaCl). Fractions of interest were collected and buffer exchanged with PBS.

For preparative gremlin purification, the heparin column was washed with PBS containing 0.6/0.5 M NaCl and then eluted with PBS containing 1.2 M NaCl. Finally, purified gremlin was buffer exchanged with PBS. Proteins were quantified by measuring the OD at 280 nm.

### Size exclusion chromatography

IMAC purified gremlin resuspended in 0.15 M sodium sulphate was loaded onto a pre-equilibrated TSK3000 PWXL column with pre-column (Tosoh Bioscieces) and subjected to 0.5 mL/min size exclusion chromatography [isocratic HPLC-GPC system (Jasko)]. Eluted fractions (0.1 mL) were collected and analysed by dot blotting using an anti-gremlin antibody (R&D system). Standard proteins (seroalbumin: 132 and 66 kDa; ovalbumin: 45 kDa; carbonic anhydrase: 29 kDa and lactoalbumin: 14,2 kDa) were used to build the calibration curve.

### Western blotting and gremlin immunoprecipitation

Samples were separated by SDS-PAGE performed under reducing [+2-MercaptoEthanol (βME)] or non-reducing (−βME) conditions. Gels were then alternatively stained by silver staining or processed for Western blot (WB) analyses with polyclonal rabbit anti-gremlin (R&D). WB/dot blot densitometry was performed with ImageJ software. For gremlin immunoprecipitation, organs were explanted from healthy C57BL/6 mice, lysed and immunoprecipitated with polyclonal goat anti-gremlin (R&D). When indicated gremlinWT or gremlinC141A were incubated in the absence or in the presence of 0.05% glutaraldehyde for 20 minutes at RT, separated by SDS-PAGE under reducing conditions and probed with polyclonal rabbit anti-gremlin.

### *In silico* docking study for gremlin homodimeric complex

Sequence alignments were obtained using CLUSTAL W software [51]. The docking software RosettaDock was used to evaluate the possible orientation and conformation of gremlin homodimers [26, 52]. We used as input the human monomeric gremlin protein structure model reshaped from the 3D structure of SOST [20]. Then, an educated guess regarding the starting position of the two monomers was uploaded in the RosettaDock query form. In this well validated approach, the algorithm identifies low-energy conformations of a protein-protein interaction near a given starting configuration by optimizing rigid-body orientation and side-chain conformations as described in [26, 52].

### Luciferase reporter gene assay

HepG2 cells were transiently transfected with pGL2-Luc-HAMP vector harbouring BMP-responsive hepcidin promoter upstream to Firefly luciferase reporter gene (kindly provided by M. Poli, Molecular Biology Laboratory, DMMT, University of Brescia), in combination with pRL-TK Renilla Luciferase vector (Promega) as a control for uniform lysate analyses. 12 hours after transfection, serum-starved cells were stimulated with 50 ng/mL of BMP4 (R&D) for 16 hours in the absence or in the presence of increasing concentrations of gremlin^WT^ or gremlin^C141A^. Cells were then lysed and luciferase activity was determined with the Dual Reporter Assay kit (Promega). Relative luciferase activity was calculated as the ratio of Firefly (reporter) to Renilla (control) luciferase activity and is expressed as percent of the activity of BMP4-stimulated cells.

### ELISA assays

96-well plates coated with 250 ng/mL of human BMP4 or seeded with VEGFR2-overexpressing ECs were incubated with gremlin^WT^ or gremlin^C141A^ in the absence or presence of VEGF-A_165_. Bound gremlin or pVEGFR2 were detected by incubation with goat anti-gremlin (R&D) or anti-pVEGFR2 (Y951; Santa Cruz Biotechnology) antibody followed by secondary horseradish peroxidase (HRP)-conjugated antibody (Santa Cruz). Peroxidase substrate (KPL, Kirkegaard & Perry Laboratories, Inc.) was finally added to the wells and absorbance was measured at 405 nm.

### BMP-antagonist assay in Zebrafish

Gremlin^WT^ and gremlin^C141A^ mRNAs were synthesized *in vitro* using the T7 mMESSAGE mMACHINE transcription kit (Ambion, ThermoFisher Scientific). 1-4 cell-stage embryos of the wild-type AB zebrafish line were injected with different amounts of mRNA and maintained in fish water at 28°C. Embryos were analysed at 22-24 hour post fertilization (hpf).

### Intracellular signalling analyses

HUVECs or VEGFR2-overexpressing ECs were treated with gremlin^WT^ or gremlin^C141A^, fixed and decorated either with anti-phospho-VEGFR2 rabbit antibody (Y1175, Cell Signaling Technology), followed by Alexa Fluor 488 anti-rabbit IgG (Invitrogen Life Technologies) or anti-phospho-Tyr mouse antibody (4G10, Upstate Biotechnology, Inc.). Cells were alternatively analysed using Axiovert 200M epifluorescence microscope equipped with Plan-Apochromat 63X/1.4 NA oil objective (Zeiss) or subjected to FACS analysis ( MACSQuant, Miltenyi Biotec) respectively. Intracellular ROS levels were assessed by measuring fluorescence intensity in HUVEC cells suspended in serum-free medium and loaded with the redox-sensitive dye DCFH-DA (5 μM, Molecular Probe, Life Technologies), as previously described [16]. Cells were then washed, stimulated with gremlin^WT^ or gremlin^C141A^ and finally subjected to FACS analysis. FACS analyses were performed with MACSQuant Analyzer (Myltenyi Biotec) and data were analysed with FlowJo software.

### HUVEC sprouting assay

Sprouting of HUVEC spheroids was analysed as described [14]. Briefly, spheroids were prepared in 20% methylcellulose medium, embedded in fibrin gel and stimulated with increasing concentrations of gremlin^WT^ or gremlin^C141A^ in the presence or the absence of VEGF-A_165_. The number of radially growing cell sprouts was counted after 24 hours using Axiovert 200M microscope equipped with LD A Plan 20X/0.30PH1 objective (Zeiss).

### CAM assay

Alginate beads containing PBS or gremlin^WT^ or gremlin^C141A^ were placed on the CAM of fertilized white Leghorn chicken eggs at day 11 of incubation [16]. After 72 hours, newly formed blood vessels converging toward the implant were counted at X5 magnification using a STEMI SR stereo-microscope equipped with an objective *f* of 100 mm with adapter ring 475070 (Zeiss).

### Matrigel plug assay

All the procedures involving mice and their care conformed to institutional guidelines that comply with national and international laws and policies (EEC council Directive 86/609, OJ L 358, 12 December 1987). 7-week-old C57BL/6 mice (Charles River Laboratories International, Inc.) were injected subcutaneously with 400 μL of liquid Matrigel (Trevigen, Inc.) containing 400 ng of gremlin. For the competition assay mice were injected with 400 μL of liquid Matrigel containing 400 ng of human VEGF-A_165_ in the absence or the presence of 2 μg of gremlin^WT^ or gremlin^C141A^. Matrigel with PBS alone was used as negative control. One week after injection, mice were sacrificed and plugs were harvested and processed for immunofluorescence using anti CD31 antibodies and RT-qPCR as previously described [53]. Matrigel slices were analysed using Axiovert 200M epifluorescence microscope equipped with Plan-Neofluar 20X/0.5 NA objective (Zeiss). CD31^+^ area was measured by ImageJ software analysis. The mRNA expression levels of murine *cd31* were normalized to the levels of human *gapdh*. Data are expressed as relative expression ratios (ΔΔCt – Fold increase) using one PBS plug as reference.

### Surface plasmon resonance (SPR) analysis

SPR measurements were performed on BIAcore X instrument (GE Healthcare). The extracellular domain D_1-7_ of human VEGFR2 (ECD-VEGFR2) (ReliaTech GmbH) was immobilized at approximately 0.036-0.064 pmol/mm^2^ onto CM5 sensorchips (BIAcore). The chips were pre-activated with a mixture of 0.2 M N-ethyl-N′-(3-dimethylaminopropyl)-carbodiimide hydrochloride and 0.05 M N-hydroxysuccinimide (35 μL; flow rate: 10 μL/minute). After ECD-VEGFR2 immobilization (70 μL of a solution of 0.345 μM sVEGFR2 in 10 mM sodium acetate pH 4.5 at flow rate 10 μL/minute), the remaining dextran active moieties were deactivated with 1.0 M ethanolamine at pH 8.5 (35 μL, flow rate 10 μL/minute). The activated/deactivated dextran was used as reference. Increasing concentrations (from 100 ng/mL to 4 μg/mL) of gremlin^WT^ or gremlin^C141A^ were injected in HBS-EP buffer (BIAcore) for 10 minutes (sample volume: 50 μL; flow rate: 5 μL/minute; dissociation time: 2 minutes). The response (in response units) was monitored as a function of time. For each concentration of the ligand, the SPR response at equilibrium was used to build the normalized dose-response binding isotherms of gremlin with ECD-VEGFR2. Binding isotherm points were fitted to the Langmuir equation for monovalent binding to determine equilibrium affinity constants, by using SOLAR2.0 software (http://www.chem4tech.it/).

### Fluorescence resonance energy transfer (FRET) analysis

FRET experiments were performed as previously described [17, 54]. Briefly, HEK293T cells, co-transfected with a pcDNA3 vector harbouring Enhanced Yellow Fluorescent Protein (EYFP)-ECD-VEGFR2 cDNA and a pcDNA3 vector harbouring Enhanced Cyan Fluorescent Protein (ECFP)-ECD-VEGFR2 cDNA, were seeded in 8-well μ-Slides coverslips (ibidi GmbH) and stimulated with 100 ng/mL of gremlin^WT^ or gremlin^C141A^ for 10 minutes. Then cells were fixed and subjected to FRET analysis. To this purpose, a region of interest was selected and photo-bleached by applying 100% intensity of a 514 nm laser. FRET efficiency was calculated using the formula: FRET = (D_post_ − D_pre_)/D_post_, where D_post_ and D_pre_ represent the donor (ECFP) emission intensities before and after photo-bleaching, respectively. FRET efficiency was also measured in a non-photobleached region of the same cell as in situ control. In all experiments, cells transfected with ECFP-EYFP fusion protein were used as FRET positive controls [55]. Pixel-pixel FRET efficiency by acceptor photobleaching was also calculated by “FRETcalc v5.0” of ImageJ Software [56].

### VEGFR2 internalization assay

HUVECs surface biotinylation was performed as previously described [57]. Briefly, serum-starved HUVECs were stimulated for 15 minutes at room temperature with gremlin^WT^ or gremlin^C141A^. Cells were then incubated for 2 hours at 4°C with biotin-3-sulfo-N-hydroxy-succinimide ester sodium salt (biotin-NHS) (Sigma) dissolved in Hanks' Balanced Salt Solution at 0.5 mg/mL. Biotin-NHS was inactivated by incubation with 50 mM Tris-HCl (pH 7.4) containing 150 mM NaCl. Cells were then lysed, immunoprecipitated with anti-VEGFR2 antibody (Santa Cruz Biotechnology) and separated by reducing SDS-PAGE. Analyses of the biotinylated immunocomplexes were performed using HRP-conjugated streptavidin (GE Healthcare).

### *In vivo* tumorigenesis assay

All the procedures involving mice and their care conformed to institutional guidelines as previously indicated. 5×10^5^ mock- or gremlin^WT^- or gremlin^C141A^-EO771 cells were injected orthotopically in 15-week-old female C57BL/6 mice (6 mice/group). Alternatively 5×10^5^ mock- or gremlin^C141A^-TRAMP-C2 cells were injected subcutaneously in 15-week-old male C57BL/6 mice (6 mice/group). At the end of experiment tumors were harvested and processed for RT-qPCR or immunofluorescence analysis. For RT-qPCR analyses, the mRNA expression levels of murine *vegf*, *vegfr2* and *ve-cadherin* were normalized to the levels of murine *gapdh*. Data are expressed as relative expression ratios (ΔΔCt – Fold increase) using one mock plug as reference. For immunofluorescence analyses, paraffin embedded tumors were stained with anti-CD31 antibody followed by incubation with Alexa Fluor 594-conjugated secondary antibody. Tumor slices were analysed using Axiovert 200M epifluorescence microscope equipped with Plan-Neofluar 20X/0.5 NA objective (Zeiss). CD31^+^ area was measured by ImageJ software analysis.

### Data representation and statistical analyses

Data are expressed as mean ± SEM. Statistical analyses were performed using One-Way ANOVA followed by Bonferroni's test or Student's *t*-test. The indicated p value was set as statistically significant.
